# Molecular data reveal a new species of *Rhopalias* Stiles & Hassall, 1898 (Digenea, Echinostomatidae) in the Common opossum, *Didelphismarsupialis* L. (Mammalia, Didelphidae) in the Yucatán Peninsula, Mexico

**DOI:** 10.3897/zookeys.854.34549

**Published:** 2019-06-10

**Authors:** Jorge López-Caballero, Rosario Mata-López, Gerardo Pérez-Ponce de León

**Affiliations:** 1 Departamento de Biología Evolutiva, Facultad de Ciencias, Universidad Nacional Autónoma de México. Avenida Universidad 3000, Ciudad Universitaria, C. P. 04510; Mexico City, Mexico Universidad Nacional Autónoma de México Mexico Mexico; 2 Departamento de Zoología, Instituto de Biología, Universidad Nacional Autónoma de México. Avenida Universidad 3000, Ciudad Universitaria, A. P. 70–153, C. P. 04510; Mexico City, Mexico Universidad Nacional Autónoma de México Mexico Mexico

**Keywords:** DNA, integrative taxonomy, phylogeny, Trematoda

## Abstract

A new species of *Rhopalias* Stiles & Hassall, 1898 is described from the small intestine of the Common opossum, *Didelphismarsupialis* Linnaeus from the Yucatán Peninsula, Mexico. *Rhopaliasoochi***sp. nov.** is morphologically very similar to the type species of the genus, *Rhopaliascoronatus* (Rudolphi, 1819) Stiles & Hassall 1898, a species widely distributed in opossums across Mexico. A molecular phylogenetic analysis using a mitochondrial gene (cox1), and the nuclear ribosomal internal transcribed spacer region (ITS1-5.8S-ITS2), of specimens of *R.coronatus* collected in several localities of Mexico revealed that those from the Yucatán Peninsula, originally recorded on morphological grounds as *R.coronatus* actually represented an independent genetic lineage. Maximum Likelihood and Bayesian Inference analyses were performed for each data set independently, and for the concatenated data set (ITS1-5.8S-ITS2 + cox1). All phylogenetic analyses showed that the specimens from Yucatán represented a monophyletic lineage, with high bootstrap support and Bayesian posterior probabilities. In addition, the genetic divergence estimated between *R.oochi***sp. nov.** and two species of *Rhopalias*, *R.coronatus*, and *R.macracanthus* Chandler, 1932 that also occur in Mexican marsupials ranged between 7–8% and 16–17%, for cox1, and between 0.1–0.2% and 7% for the ITS region, respectively. The molecular evidence gathered in this study (reciprocal monophyly in both phylogenetic analyses, and estimated genetic divergence) suggested that the specimens found in the intestine of *D.marsupialis* originally reported as *R.coronatus* from Yucatán, actually represent a new species. Morphological evidence was found through light and scanning electron microscopy to support the species distinction based on molecular data.

## Introduction

The genus *Rhopalias* Stiles & Hassall, 1898 includes six species of digenetic trematodes that infect the small intestine of didelphimorph marsupials of the New World (Haverkost and Gardner 2008). Members of this genus of echinostomatid trematodes (see Tkach et al. 2016) are distinguished by having two anterior tentacles armed with spines, which can be invaginated into a muscular pouch, one on each side of oral sucker (Kostadinova 2005). In a taxonomic review of the species of the genus *Rhopalias*, Haverkost and Gardner (2008) discussed the morphological characters that could be used for distinguishing among congeneric species, concluding that the number and size of tentacle spines, the presence or absence of oral and/or flanking spines, and the length of the muscular pouches are the most reliable characters. Nevertheless, they cautioned that a more extensive sampling of each species of *Rhopalias* was necessary to support the use of these characters for the species delimitation. Furthermore, with the exception of the sequence of the 28S rRNA gene from an individual of *R.macracanthus* Chandler, 1934, a parasite of the Virginia opossum, *D.virginiana* Kerr from the U.S. (Tkach et al. 2016), and genetic information about the other species of *Rhopalias* is lacking. The main objective of this study was to explore the genetic diversity among specimens of *R.coronatus* collected throughout a geographical range across southern Mexico, following a molecular prospecting approach in the search for cryptic species (sensu Blouin 2002). Molecular data were used in combination with a morphological study of newly sampled specimens of *R.coronatus*, and those deposited at the Colección Nacional de Helmintos (**CNHE**) to describe a new species of *Rhopalias*. Specimens originally recorded as *R.coronatus* from the Yucatán Peninsula by Acosta-Virgen et al. (2015) corresponded with an undescribed species. We describe the new species herein.

## Materials and methods

### Specimen sampling

**Figure 1.**
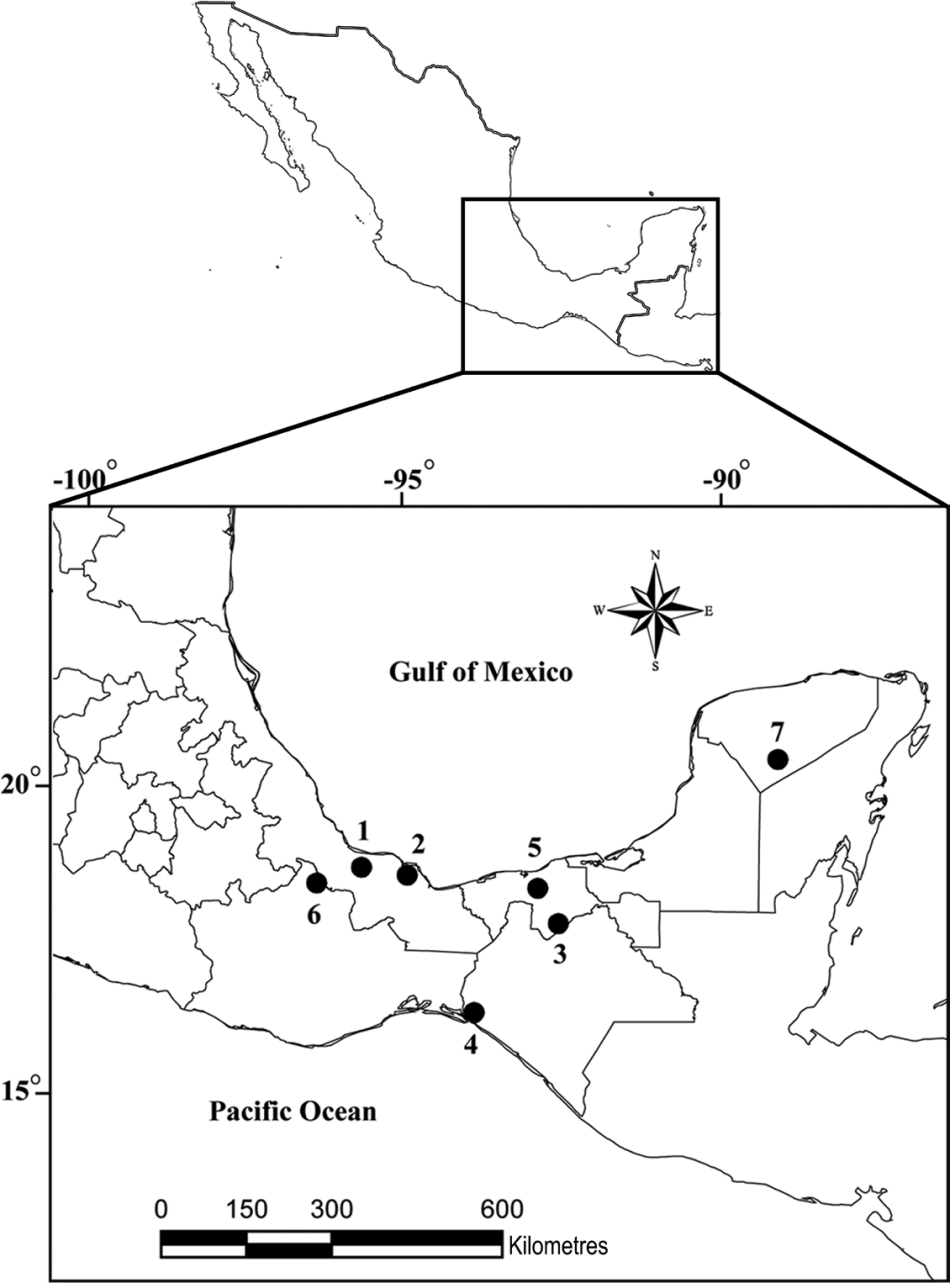
Sample collection sites of specimens of *Rhopalias* spp. in opossums across southeastern Mexico. **1** Tlacotalpan, Veracruz (TL) **2** Los Tuxtlas, Veracruz (LT) **3** Cunduacán, Tabasco (CU) **4** Agua Fría, Chiapas (AF) **5** Teapa, Tabasco (TE) **6** Soyaltepec, Oaxaca (SO) **7** Rancho Hobonil, Tzucacab, Yucatán (TZ). Numbers refer to map ID in Table 1.

In total, 44 specimens of opossums were collected between August 2011 and November 2013, in seven localities across southeastern Mexico (Fig. 1, Table 1). Hosts were collected under the collecting permit FAUT-0057 issued to GPPL by the Secretaría del Medio Ambiente y Recursos Naturales. The mammals were sacrificed with an overdose of pentobarbital sodium, necropsied, and all organs were separated in Petri dishes with 0.85% saline, and examined under a stereomicroscope. A small piece of host tissue was taken from each individual and saved for further DNA studies. These tissues are available upon request. Trematodes were removed from the intestine of their hosts, and washed in saline for 3–5 min. Some specimens were fixed by sudden immersion in hot 4% formaldehyde and stored in 70% ethanol for morphological analyses. For molecular study, eight specimens were washed with saline solution, preserved in 100% ethanol, and stored at -20 °C. These specimens were used for DNA extraction, including four specimens that were cut in half and the posterior half processed for morphology (hologenophores, sensu Pleijel et al. 2008), and four complete specimens. The four hologenophores and remaining seven specimens (paragenophores) were stained in Mayer’s paracarmine and mounted as permanent slides in Canada balsam. Specimens of *Rhopalias* were morphologically identified either as *R.coronatus* or *R.macracanthus* according with the description and morphometrical traits reported by Haverkost and Gardner (2008).

**Table 1.** Collecting localities, host species (Didelphidae) by locality, GenBank and Colección Nacional de Helmintos (CNHE) accession numbers. Map ID corresponds with numbers in Fig. 1.

Map ID	Locality (abbreviation)	Coordinates	Host (sample size)	Species	GenBank		CNHE
					cox1	ITS1-5.8S-ITS2	
1	Tlacotalpan, Veracruz (TL)	18°37'40"N, 95°40'40"W	*D.marsupialis* (5)	* R. coronatus *	MK982742–57	MK982805–06	9502, 9503
			*D.virginiana* (4)				
			*P.opossum* (2)	* R. macracanthus *	MK982783–85	MK982815	9509
2	Los Tuxtlas, Veracruz (LT)	18°34'21"N, 95°04'30"W	*D.marsupialis* (5)	* R. coronatus *	MK982674–99	MK982813–14	9499, 9500, 9501
			*D.virginiana* (1)				
			*P.opossum* (2)				
3	Cunduacán, Tabasco (CU)	17°34'17"N, 92°57'09"W	*D.virginiana* (3)	* R. coronatus *	MK982700, MK982702	–	9497
4	Agua Fría, Chiapas (AF)	16°15'26"N, 93°53'55"W	*D.marsupialis* (3)	* R. coronatus *	MK982703–41	MK982786–96	9488, 9489, 9491
			*D.virginiana* (2)				
			*P.opossum* (11)				
5	Teapa, Tabasco (TE)	17°33'49"N, 92°45'40"W	*D.marsupialis* (3)	* R. coronatus *	MK982701	MK982801	9498
6	Soyaltepec, Oaxaca (SO)	18°15'28"N, 96°24'00"W	*D.virginiana* (2)	* R. coronatus *	MK982758–74	MK982797–99, MK982807–12	9495
7	“Rancho Hobonil” Tzucacab, Yucatán (TZ)	20°00'58"N, 89°01'12"W	*D.marsupialis* (1)	*R.oochi* sp. nov.	MK982775–82	MK982800, MK982802–04	9504, 10926, 11069

### Amplification and sequencing of DNA

Individual worms fixed in 100% ethanol (or the posterior portion in some cases) were placed in tubes and digested overnight at 56 °C in a solution containing 10 mM Tris-HCl (pH 7.6), 20 mM NaCl, 100 mM Na_2_ EDTA (pH 8.0), 1% Sarkosyl, and 0.1 mg/mL proteinase K. Following digestion, DNA was extracted from the supernatant using DNAzol reagent (Molecular Research Center, Cincinnati, Ohio) according to the manufacturer’s instructions. A fragment of the mitochondrial cytochrome *c* oxidase subunit 1 (cox1), and ITS1-5.8S-ITS2 were amplified by PCR, using the primers shown in Table 2.

**Table 2.** Primers used in the present study.

Locus	Primer	Sequence (5’–3’)	Use	Reference
cox1	MplatCOX1dF	TGTAAAACGACGGCCAGTTTWCITTRGATCATAAG	PCR^†^	Moszczynska et al. 2009
	BARCOXR	ATAAACCTCAGGATGCCCAAAAAA	PCR	Razo-Mendivil (pers. comm.)
	M13F	TGTAAAACGACGGCCAGT	SEQ^‡^	Messing (1993)
(ITS1-5.8S-ITS2)	BD1	GTCGTAACAAGGTTTCGGTA	PCR & SEQ	Bowles and McManus (1993)
	BD2	TATGCTTAAATTCAGCGGGT	PCR & SEQ	Bowles et al. 1995
	BD3	GAACATCGACATCTTGAACG	SEQ	Hernández-Mena et al. 2014
	BD4	ATAAGCCGACCCTCGGC	SEQ	Hernández-Mena et al. 2014

^†^ = amplification; ^‡^ = sequencing.

All PCRs were performed at a final volume of 25 μl consisted of 2.5 μl of 10× PCR buffer, 2.5 μl of 10 mM of dNTPs mixture (200 μl each), 1.25 μl MgCl_2_ (50 mM), 1.0 μl each primer (10 pmol), 2 μl DNA template, and 1 unit of Taq DNA polymerase (Biogenica, Mexico City), and the remaining volume of sterilized distilled water. The amplification program for cox1 consisted of: initial denaturation at 96 °C for 3 min, followed by 35 cycles at 94 °C for 1 min, annealing at 50 °C for 45 s, extension at 72 °C for 1 min, followed by 10 min at 72 °C for final elongation. For the ITS1-5.8S-ITS2, the PCR conditions were: initial denaturation at 96 °C for 3 min, followed by 35 cycles at 94 °C for 1 min, annealing at 53 °C for 1 min, extension at 72 °C for 1 min, followed by a final elongation at 72 °C for 10 min. PCR products were treated with Exo–SAP–IT (Thermo Scientific), according to the manufacturer’s instructions. Cox1 and ITS1-5.8S-ITS2 products were sequenced in both strands using the primers mentioned in the Table 2, by the High Throughput Genomics Unit at the University of Washington, USA, (http://www.htseq.org./index.html). Contigs were assembled using the platform Geneious v.5.1.7 (Drummond et al. 2010). As an additional check on accuracy, cox1 nucleotide sequences were translated using Mesquite v.2.75 (Maddison and Maddison 2011), and trematode mitochondrial genetic code. All the cox1 and ITS1-5.8S-ITS2 sequences generated in this study were deposited in the GenBank (Table 1).

### Phylogenetic analyses

DNA fragments of the cox1 and ITS region were aligned separately using the software Clustal W2 (Thompson et al. 1994) with a final manual adjusting in Mesquite v.2.75. The concatenated data set was aligned using the same software. Sequences of cox1 and ITS region of other species included within superfamily Echinostomatoidea Looss, 1899 available in the GenBank were used as outgroups. Maximum Likelihood (ML) and Bayesian Inference (BI) analyses were performed for each data set, and for the concatenated data set (ITS1-5.8S-ITS2 + cox1) partitioned by gene. The program jModeltest v.3.0 (Posada and Crandall 1998) was used for inferring the best model of evolution for each data set using the Akaike information criterion. The TVM + I + G and TPMuf + G substitution model were the best models for cox1 and ITS region, respectively. The ML trees were inferred using RAxML v.7.0.4 (Stamatakis 2006). Bootstrap resampling with 10,000 replicates assessed ML clade support. Additionally, Bayesian analyses were performed with the program MrBayes v.3.2.1 (Ronquist et al. 2012). The settings were two simultaneous runs with four Markov chains Monte Carlo (MCMC) for 10 million generations, sampling every 200 generations, a heating parameter value of 0.2 and a ‘burn-in’ of 10%. A 50% majority-rule consensus tree representing the posterior probability distribution of clades was produced of the sampled trees. Phylogenetic trees were displayed with the program FigTree v.1.4.2 (Rambaut 2006). Finally, genetic divergence (p-value) was calculated for each data set using MEGA v.6.0 (Tamura et al. 2013).

### Morphological analyses

Representative specimens of the species of *Rhopalias* were stained with Mayer’s paracarmine or Gomori’s trichrome, dehydrated through a graded ethanol series (70%, 80%, 90% [twice], and 100%), cleared in methyl salicylate, and mounted in Canada balsam. The specimens were observed using an Olympus BX81 light microscope. Some worms were drawn with the aid of a drawing tube attached to an Olympus BX53 light microscope. Likewise, in order to obtain a complete digital record of the morphological traits, specimens were observed through the Differential Interference Contrast method (DIC), using an Olympus Provis AX70 microscope. Photomicrographs of the specimens were obtained with a digital camera Evolution 5.0 MP. All specimens were measured using the software Image Pro-Plus v.7.0. Measurements are presented in micrometres (μm) unless otherwise stated. For scanning electron microscope studies (SEM), the specimens were dehydrated in a graded series of alcohol solutions and then critical point dried with carbon dioxide. Specimens were mounted on metal stubs with carbon adhesive, and then gold coated and examined at 15kV in a Hitachi Stereoscan Model SU1510 SEM (Hitachi Ltd., Tokyo, Japan). Digital images of these specimens were obtained using digital imaging software attached to a computer. Specimens of the new species of *Rhopalias* were deposited in the Colección Nacional de Helmintos (**CNHE**), Instituto de Biología, Universidad Nacional Autónoma de México, Mexico City (Table 1).

## Results

### Phylogenetic analysis

**Figure 2.**
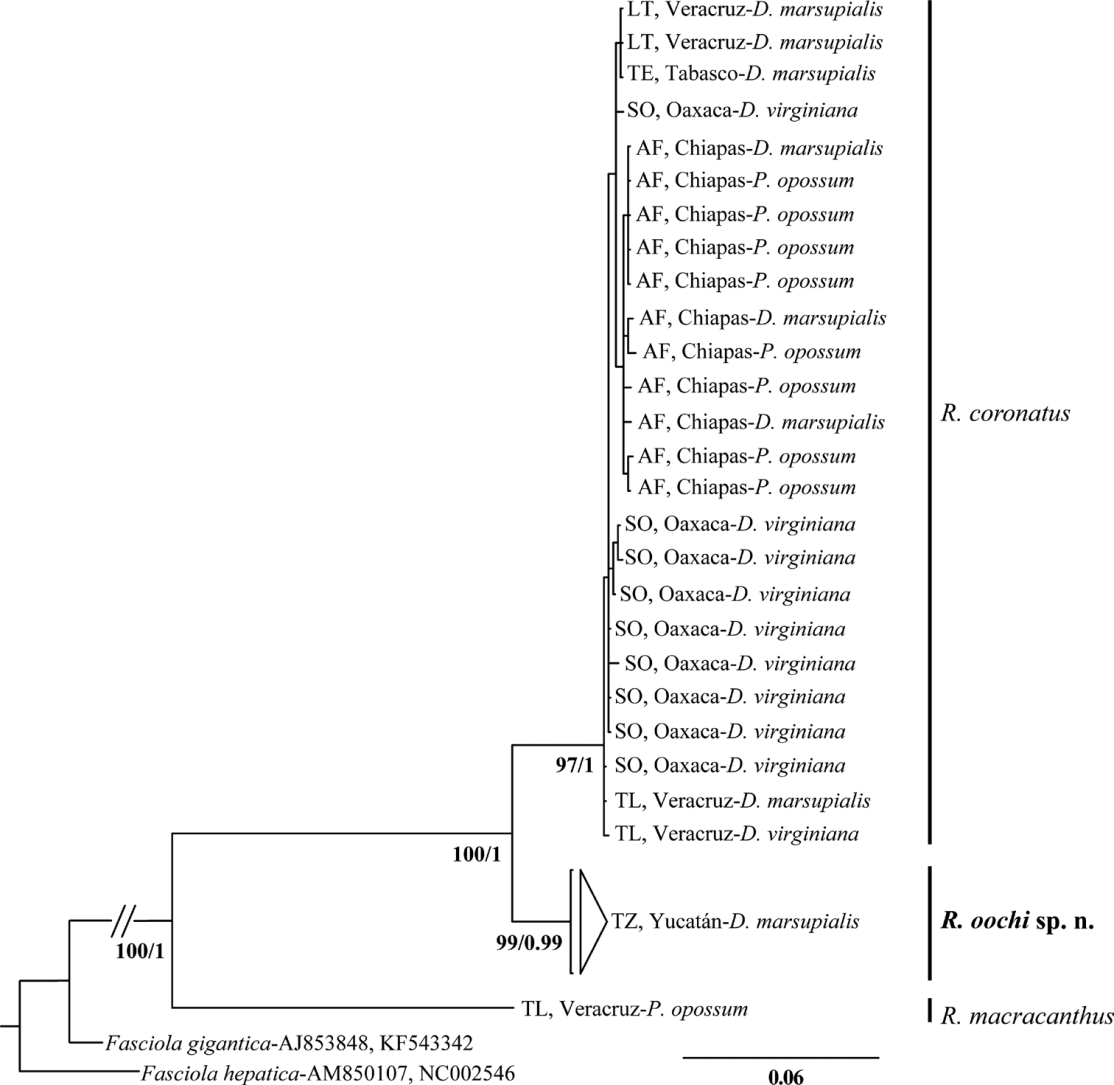
Tree inferred with the concatenated data set (ITS1-5.8S-ITS2 + cox1), using Maximum Likelihood (-ln likelihood 4605.087967), and Bayesian Inference. The numbers at the nodes represent ML bootstrap support and posterior probability values. Terminals show the locality (abbreviation as in Table 1), and host species where each isolate was collected.

**Cox1.** One hundred twelve sequences of cox1 were obtained in the present study, including 101 of *R.coronatus*, three of *R.macracanthus*, and eight of the new species. The final alignment included seven sequences from GenBank (as outgroups), and consisted of 119 sequences with 666 bp. Phylogenetic trees reconstructed by ML and BI yielded similar topologies with high bootstrap support and Bayesian posterior probabilities, respectively (see Suppl. material 1: Figure S1). The tree obtained with both reconstruction methods shows three major clades, the same ones that were obtained in the concatenated data set (Fig. 2). Clade I, corresponding to *R.coronatus* according to their morphology, included 101 sequences from six localities: 26 isolates from Los Tuxtlas (LT), 16 isolates from Tlacotalpan (TL), 17 from Soyoltepec (SO), 39 from Agua Fría (AF), two isolates from Cunduacán (CU) and one from Teapa (TE). Clade II included eight sequences from a single locality: Rancho Hobonil, Tzucacab (TZ), representing the new species. Finally, Clade III was composed by three isolates from Tlacotalpan (TL), which were morphologically determined as *R.macracanthus*. This last clade was recovered as the sister group of clades I and II (see Suppl. material 1: Fig. S1).

**ITS.** A subsample of 30 sequences of ITS1-5.8S-ITS2 region were obtained in this study from some specimens corresponding with each of the three genetic lineages of *Rhopalias* from the cox1 analysis; samples included 25 individuals of *R.coronatus*, one of *R.macracanthus*, and four sequences of the new species. In addition, ITS sequences of other species included in the superfamily Echinostomatoidea were downloaded from GenBank and used as outgroups. The final alignment consisted of 32 sequences with 1093 bp. Phylogenetic analyses by ML and BI yielded the same topology (see Suppl. material 2: Figure S2). Both topologies showed two major clades (and not three as the cox1 tree), and relationships are supported by high bootstrap and posterior probability values. Clade I included 29 sequences from six localities: two isolates from Los Tuxtlas (LT), two isolates from Tlacotalpan (TL), nine from Soyoltepec (SO), eleven from Agua Fría (AF), one from Teapa (TE) and four isolates from Tzucacab (TZ). However, these four sequences formed a small subclade within Clade I, corresponding with the isolates of the new species; Clade II was composed by only one sequence of *R.macracanthus* from Los Tuxtlas (LT).

**Concatenated data set.** This data set consisted of ITS1-5.8S-ITS2 plus the cox1 gene sequences. The final alignment included 32 sequences with 1759 bp. Phylogenetic analyses of this concatenated data set were also conducted using ML and IB methods, yielding the same topologies. The ML tree is shown in Figure 2. The tree yielded three major clades well supported by bootstrap and Bayesian posterior probability values. The first one included all samples of *R.coronatus*: two specimens from Los Tuxtlas (LT), two specimens from Tlacotalpan (TL), nine specimens from Soyoltepec (SO), eleven specimens from Agua Fría (AF), and one from Teapa (TE). The second clade included four isolates from Rancho Hobonil, Tzucacab (TZ), representing the new species. Clade III included only one sequence from Tlacotalpan (TL), corresponding with *R.macracanthus*. All the individuals sequenced from each of these three *Rhopalias* species, sampled in most of their geographic distribution in Mexico, formed monophyletic groups, with *R.coronatus* and the new species grouped as sister species (Fig. 2).

**Genetic divergence.** The genetic divergence estimated among populations of the new species with respect to *R.coronatus* and *R.macracanthus* ranged between 7–8% and 16–17%, for cox1, respectively; for the internal transcribed spacers, interspecific divergence between the new species and the other two species of *Rhopalias* varied 0.1–0.2% and 7%, respectively. The intraspecific divergence among isolates of the three species of *Rhopalias* ranged from 0–1% for cox1, was null for ITS.

### Family Echinostomatidae Looss, 1899

#### Genus *Rhopalias* Stiles & Hassall, 1898

##### Rhopalias oochi sp. nov.

Taxon classificationAnimaliaPlagiorchiidaEchinostomatidae

http://zoobank.org/2AFA9155-52CE-4436-A95F-2B0E85F93C72

**Figure 3.**
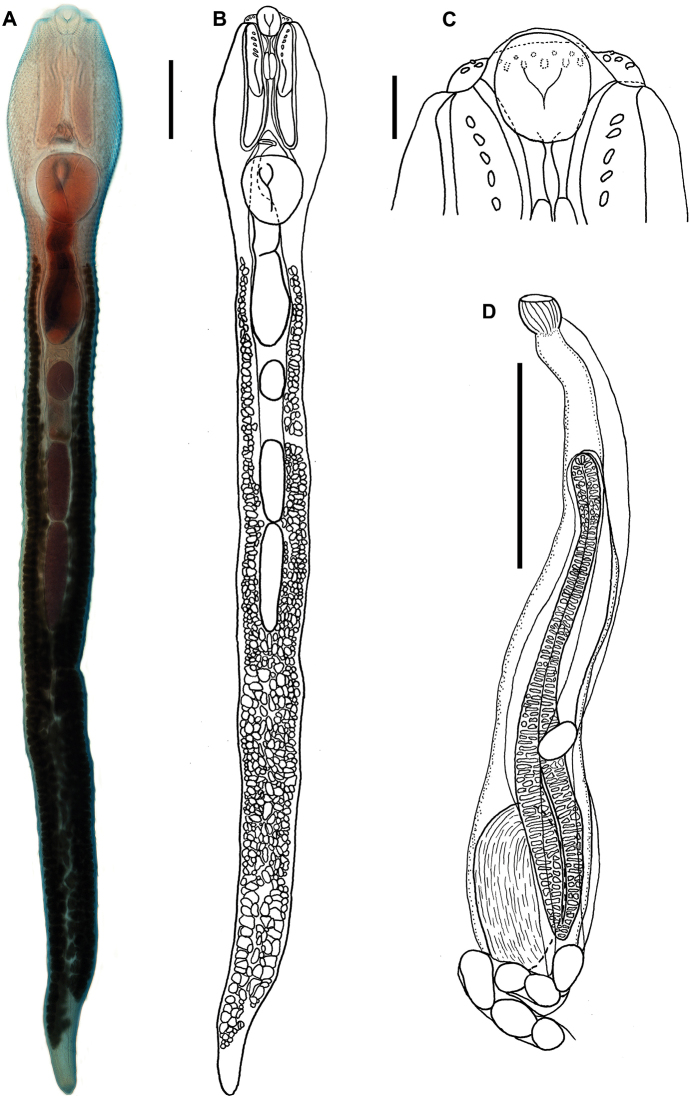
*Rhopaliasoochi* sp. nov., parasite of *Didelphismarsupialis* from Yucatán, Mexico. **A** Microphotograph, ventral view of entire body **B** Line drawing, ventral view **C** Detail of the oral, flanking and tentacle spines, ventral view **D** Cirrus sac, vagina, and eggs, ventral view. Scale bars: 10 μm (**A–C**); 400 μm (**D**).

**Figure 4.**
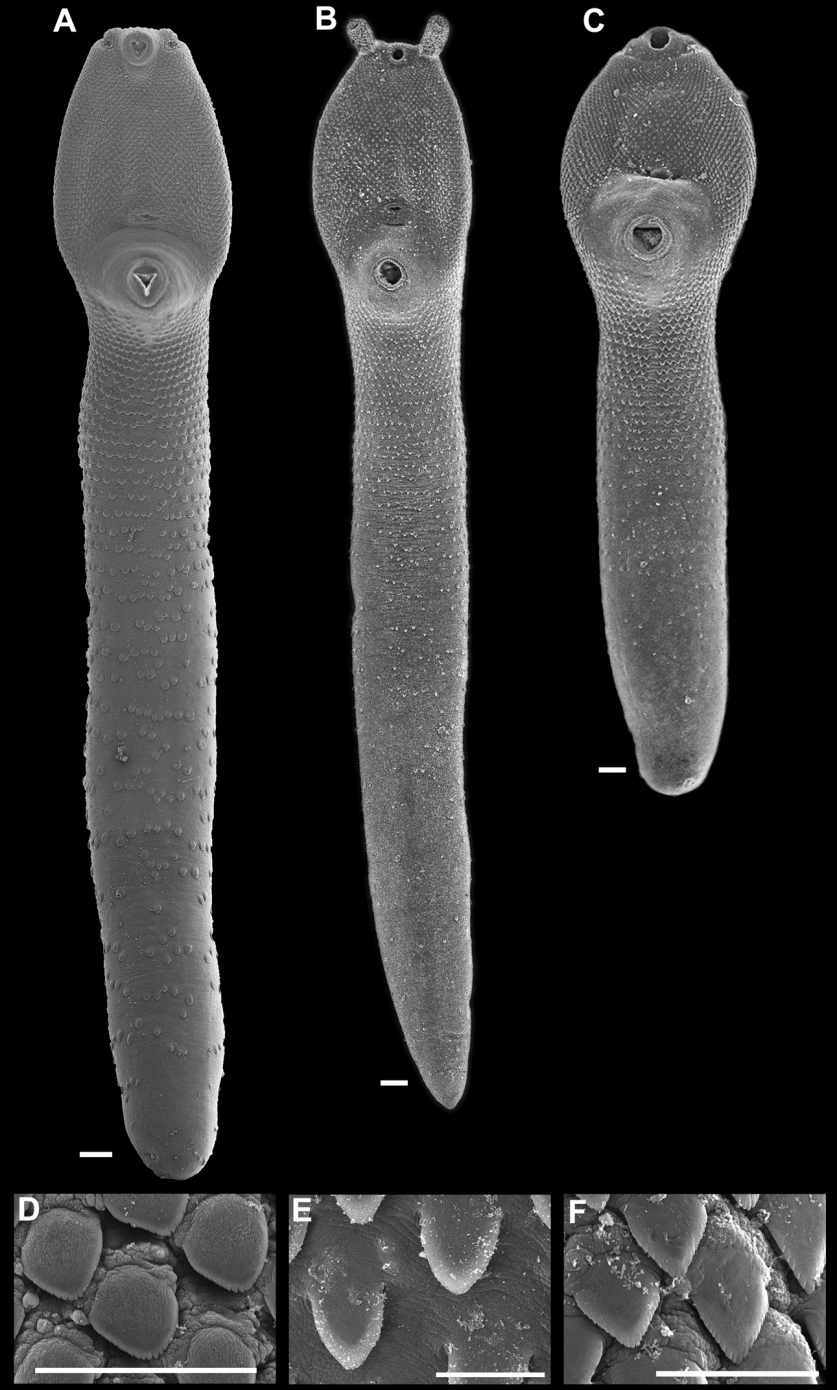
SEM micrographs of *Rhopalias* species. **A–C** Ventral view of the body of adult specimens showing the distribution of spines **D–I** Detail of the spines shape on the ventral surface of hindbody **A, D, E***R.oochi* sp. nov. **B, F, G***R.coronatus***C, H, I***R.macracanthus*. Scale bars: 100 μm (**A–C**); 50 μm (**D, E**); 10 μm (**F, G**); 20 μm (**H, I**).

**Figure 5.**
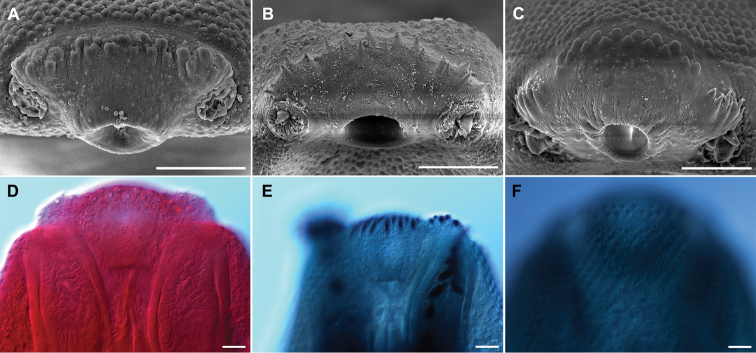
Anterior end of *Rhopalias* species. showing the detail of the oral and flanking spines. **A–C** SEM micrographs **D–F** DIC micrographs, dorsal view **A, D***R.oochi* sp. nov. **B, E***R.coronatus***C, F***R.macracanthus*. Scale bars: 100 μm (**A, B**); 50 μm (**C**); 40 μm (**D–F**).

Figures 3A–D , 4 A, D, E , 5A, D

###### Synonym.

*Rhopaliascoronatus* of Acosta-Virgen et al. (2015). Specimens deposited in the CNHE (9504).

###### Type host.

*Didelphismarsupialis* Linnaeus, Common opossum (Mammalia: Didelphidae).

###### Type locality.

Rancho Hobonil, Tzucacab, Yucatán state, Mexico (20°00'58"N, 89°01'12"W).

###### Site in host.

Small intestine.

###### Prevalence and intesity of infection.

100% (1 of 1 opossum), infected with 15 trematodes.

###### Type specimens.

Holotype: CNHE 9504; paratypes: CNHE 10926 (3 specimens) and hologenophores CNHE 11069 (4 specimens).

###### Etymology.

The specific epithet refers to the common name of the host where the new species was found. In the Mayan language, “ooch” means opossum.

###### Description.

Based on 11 adult specimens (including 4 hologenophores). Measurements are given in Table 3. Trematodes with a long body, forebody concave, wider than hindbody, with a pair of armed retractile tentacles with 4–7 spines (Fig. 3A, B, C). Tegument covered with spines reaching posterior end of body (Fig. 4A). Tegument spines U-shaped, with distal tip pectinated (Fig. 4D, E). Oral and flanking spines present (Figs 3C, 5A, 5D). Muscular sacs long, reaching far beyond posterior margin of pharynx, and may or may not reach the anterior margin of ventral sucker (Fig. 3A, B). Oral sucker subterminal, rounded, well-developed, short prepharynx, pharynx muscular, and relatively long oesophagus; caecal bifurcation at short distance anterior to genital pore (Fig. 3B); long caeca extending to the posterior end of body (Fig. 3A). Ventral sucker muscular and subspherical, in the first third of body, larger than oral sucker (Figs 3A, B, 4A). Testes two, elongated, in tandem, contiguous, no overlapped, located in mid-body; anterior testis shorter than posterior testis (Fig. 3B). Cirrus sac long, claviform, containing a well-developed prostate complex and seminal vesicle, extending beyond ventral sucker and terminating near anterior border of ovary. Genital pore between ventral sucker and caecal bifurcation. Ovary slightly oval, postacetabular, pretesticular. Uterus intercaecal, between ovary and genital pore. Metraterm long (Fig. 4D). Vitelline follicles in lateral fields, beginning at mid-level between ventral sucker and ovary, ending at posterior end. Gravid specimens with few eggs, oval-shaped, operculated; embryonated eggs with thin shell.

###### Remarks.

The genus *Rhopalias* currently contains six species as parasites of New World marsupials (Haverkost and Gardner 2008), i.e., *Rhopaliascoronatus*, the type species; *Rhopaliashorridus* (Diesing, 1850) Stiles and Hassall 1898; *Rhopaliasbaculifer* Braun, 1901; *Rhopaliasmacracanthus* Chandler, 1932; *Rhopaliascaballeroi* Kifune & Uyema, 1982; and *Rhopaliascaucensis* Rivillas, Caro, Carvajal & Vélez, 2004. *Rhopaliasoochi* sp. nov. represents the seventh described species and is readily distinguished from five of the congeneric species, excepting *R.coronatus* by having tentacle sacs extending far beyond the posterior margin of pharynx. In their detailed morphological revision of the genus *Rhopalias*, Haverkost and Gardner (2008) provided a key to species of the genus and found that this character is reliable to distinguish between two groups of species. In *R.horridus*, the tentacle sacs surpass the posterior margin of pharynx to reach the mid-level of oesophagus; however, they never extend beyond the caecal bifurcation. In *R.oochi* sp. nov. the tentacle sacs extend to reach the anterior margin of ventral sucker. *Rhopaliashorridus* further differ from the new species, and the remaining congeners by the large number of tentacle spines (> 30) (see key in Haverkost and Gardner 2008). The new species most closely resembles the type species, *R.coronatus*; in fact the specimens upon which the description of the new species is based were originally recorded as *R.coronatus* (see Acosta-Virgen et al. 2015). Genetic data revealed that they might actually represent a different species. In *R.coronatus* as in the new species tentacle sacs extend far beyond the posterior border of pharynx; in *R.coronatus*, both sacs usually surpass the anterior border of ventral sucker (although our observation of numerous voucher specimens indicate that the extension is variable among individuals); in the new species, the tentacle sacs consistently extend to almost reach the anterior border of ventral sucker, but never surpass it. Additionally, *R.coronatus* differ from the new species by having larger oral spines (at least four times larger), and by the distribution of vitelline follicles. In *R.coronatus* follicles extend between the posterior end of body and the posterior border of ventral sucker; instead, in *R.oochi* sp. nov. vitelline follicles do not reach the posterior end of body, and in the forebody, they reach the mid-level of cirrus sac. Morphometrically, most measurements are clearly overlapped between the new species and *R.coronatus* (except in oral spines size). We observed and measured 21 individuals of *R.coronatus* deposited at the CNHE. Our measurements, and those reported in other studies (see Table 3) indicate that *R.coronatus* is morphometrically quite variable. Furthermore, SEM study of the ultrastructure of the body surface provided additional evidence to distinguish the new species from *R.coronatus* and *R.macracanthus*; for these two species we collected specimens and some were fixed for a comparative SEM study (Fig. 4). In the three species body spines are denser in the forebody; however, the extension, size, and shape of spines clearly distinguish the three species. In *R.oochi* sp. nov., spines are robust and pectinate and they extend along the body almost to the posterior end (Fig. 4A, D, E); meanwhile, in *R.coronatus* spines are distributed to the mid-level of hindbody and are entire and tongue-shaped (Fig. 4B, F, G) and in *R.macracanthus*, even though spines extend more posteriorly, they are not as robust as in the new species; spines in *R.macracanthus* are pectinate and arrow-shaped (Fig. 4C, H, I).

## Discussion

Specimens of *Rhopalias* studied in the present study were identified morphologically either as *R.coronatus* or *R.macracanthus* following the descriptions by Haverkost and Gardner (2008). Identification was based on the length of the muscular sacs. The species *R.coronatus* possess muscular sacs almost reaching the anterior end of ventral sucker; meanwhile in *R.macracanthus* muscular sacs are short and slightly overpass the pharynx level. Additionally, spines of the retractile tentacles are very large in *R.macracanthus* (see Suppl. material 3: Figure S3C) in comparison with those of *R.coronatus*. Morphometrically, specimens of the three species are also different (see Table 3). Our study followed a molecular prospecting approach by considering *R.coronatus* as a trematode species that infects three species of marsupials distributed across a wide geographical range in southeastern Mexico (Blouin 2002; Criscione et al. 2005; Vilas et al. 2005). Our analyses showed that the specimens from the Yucatán Peninsula actually represented a separate species; phylogenetic trees showed all isolates from that locality as a reciprocally monophyletic assemblage, separated from isolates of *R.coronatus*, and this two as the sister taxa of *R.macracanthus*. Even though the internal transcribed spacers (ITS1-5.8S-ITS2) exhibited lower resolution, the mitochondrial gene (cox1) revealed relatively high genetic divergence values (7–8%) supporting the distinction of the new species. This level of genetic divergence has been found in studies with other members of the superfamily Echinostomatoidea. For instance, Saijuntha et al. (2011) reported divergence levels of 8–16% between two species of *Echinostoma* Rudolphi, 1809. Even though the pertinence of using a genetic yardstick to distinguish parasite species has been questioned (Nadler and Pérez-Ponce de León 2011), the species delimitation criteria followed in our study is also based on a hypothesis-testing framework (see Adams 2002; Nadler 2002).

**Table 3.** Measurements of *Rhopalias* spp. Measurements are presented in micrometers (µm) unless otherwise noted. Measurements above 1000 µm are expressed in millimeters.

	*R.oochi* sp. nov. (Present study) N= 7			*R.coronatus* (Present study) N= 15			*R.coronatus* (Haverkost & Gardner 2008) N= 22			*R.macracanthus* (Present study) N= 6		
	n	Mean	Range	n	Mean	Range	n	Mean	Range	n	Mean	Range
Body L	4	6.64 mm	6.42–6.70	15	4.69	2.94–6.66	22	4.440	2.160–9.360	6	3.36	2.78–3.75
Body W	4	727	622–810	15	611	320–770	22	735	219–1.58	6	680	514–680
VS L	7	484	420–543	15	330	190–530	22	376	150–840	6	308	257–340
VS W	7	417	355–464	15	330	190–480	22	350	150–816	6	304	273–332
OS L	6	177	168–189	15	180	100–245	22	183	93–344	6	159	141–174
OS W	6	160	139–194	15	167	99–200	22	180	88–325	6	162	149–174
Cirrus sac L	6	1.30	1.14–1.43	15	931	332–1.50	22	970	563–2.219	4	960	871–1.03
Cirrus sac W	6	283	248–317	15	281	132–400	20	203	119–500	4	319	174–431
Anterior testis L	4	528	500–572	15	386	255–515	21	333	156–625	6	277	195–356
Anterior testis W	4	149	146–154	15	189	135–227	21	167	100–281	6	220	130–350
Posterior testis L	4	722	672–770	15	535	322–655	21	499	256–919	5	412	299–520
Posterior testis W	4	140	137–147	15	175	112–232	21	149	75–281	5	164	123–217
Ovary L	5	222	112–233	14	175	75–232	21	169	75–344	6	151	97–175
Ovary W	5	178	162–193	14	162	95–217	20	178	88–350	6	149	86–180
Tentacle sac L	7	741	654–798	14	710	423–990	21	693	375–1.188	6	301	271–347
Tentacle sac W	7	155	137–189	14	146	109–200	21	137	63–238	6	132	125–143
Prepharynx L	5	103	90–118	15	80	30–120	22	39	0–313	6	78	56–97
Pharynx L	6	173	151–212	15	156	105–200	22	202	115–425	6	162	147–182
Pharynx W	6	96	84–111	15	81	47–135	21	104	30–244	6	99	91–112
Oesophagus L	4	312	300–339	15	285	200–422	20	186	0–606	6	19	10–30
Egg number	5	6	0–12	15	30	22–90	22	24	0–75	6	25	10–75
Eggs L	12	83	66–96	58	89	70–113	94	90	70–108	30	86	60–100
Eggs W	12	51	41–55	58	50	30–62	94	51	38–70	30	54	39–93
VS/OS ratio L	6	1:2.70	1:2.60–2.87	15	1:1.83	1:1.90–2.20		1:2.0*	1:1.61–2.44*	6	1:1.93	1:1.82–1.95
VS/OS ratio W	6	1:2.70	1:2.60–2.80	15	1:1.00	1:1.90–2.40		1:1.94*	1:1.70–2.50*	6	1:1.87	1:1.83–1.90
ANTVIT	4	1.66 mm	1.56–1.75	15	1.31	715–2.72	22	1.16	331–3.60	6	1.10	1.05–1.20
VSVIT	4	248	176–328	14	38	-130–237	22	51	-200–480	6	40	0–10
GP to anterior end	5	821	795–853	15	660	430–760		NM	NM	6	525	430–589
Oral spines L	14	9	7–12	30	39	22–58		NM	NM	24	10	12–20
Oral spines W	14	8	5–10	30	11	7–14		NM	NM	24	12	7–17
Tentacle spines L	18	23	15–32	30	36	33–55	22	56	32–67	24	135	112–152
Tentacle spines W	18	11	8–14	30	19	14–30		NM	NM	24	25	20–32

VS = Ventral Sucker; OS = Oral Sucker; ANTVIT = distance from the anterior end to the anterior margin of the vitellarium; VSVIT = distance from the anterior margin of the vitellarium to the posterior margin of the Ventral Sucker; GP = Genital Pore; NM = No mentioned; * = Values estimated from original measurements in Haverkost and Gardner (2008).

A closer look at the morphology of the specimens from Yucatán using light and scanning electron microscopy corroborated the molecular results, and the new species was described as a parasite of the Common opossum, *D.marsupialis*. *Rhopaliasoochi* sp. nov. represents the seventh described species in the genus, and the 5^th^ in marsupials distributed in Mexico. With the exception of *R.macracanthus*, a species described by Chandler (1932) in the Nearctic biogeographical region, from the Virginia opossum, in the U.S.A., all the other species included in the genus *Rhopalias* were originally described in marsupials from the Neotropical region. However, *R.macracanthus* was later found in South American marsupials, particularly in the Department of Santa Cruz, Bolivia (Haverkost and Gardner 2008). The type species, *R.coronatus* was described from the Common opossum, *D.marsupialis* in Brazil; *R.horridus* from the Water opossum, *Chironectesminimus* (Zimmerman) also in Brazil; *R.baculifer* from *D.marsupialis* in Brazil; *R.caballeroi* from *D.marsupialis* and from the Grey four-eyed opossum, *Philanderopossum* (Linnaeus) in Brazil; and *R.caucensis* from *P.opossum* in Colombia (see Haverkost and Gardner 2008). According to García-Prieto et al. (2012) four species of *Rhopalias* have been recorded in Mexico, *R.baculifer* and *R.caballeroi* in restricted localities in the tropical rain forest of Los Tuxtlas, Veracruz, and other two species more widely distributed parasitizing three species of marsupials (*P.opossum*, *D.virginiana* and *D.marsupialis*), *R.coronatus*, and *R.macracanthus* in nine and 12 localities, respectively. A few additional records were more recently provided by Acosta-Virgen et al. (2015). Interestingly, in the region of Los Tuxtlas, Veracruz, where the three species of opossums are found in sympatry (see Cervantes et al. 2010), the four species of *Rhopalias* have been recorded. In our study, even though we sampled five individuals of *D.marsupialis*, one of *D.virginiana* and two of *P.opossum* from the same locality, we only collected specimens of *R.coronatus* and *R.macracanthus*.

Marsupials occurring across Mexico are heavily parasitized by helminths; at least 16 helminth taxa have been recorded for *D.marsupialis*, 30 for *D.virginiana*, and 17 for *P.opossum* across their distributional ranges in Mexico (Acosta-Virgen et al. 2015). The only study where DNA sequences were used to establish a more robust species delimitation for the helminth parasite fauna of marsupials is that of López-Caballero et al. (2015). These authors uncovered three genetic lineages for the acanthocephalan *Oligacanthorhynchusmicrocephalus* (Rudolphi, 1819) Schmidt, 1972, a species allegedly with a distribution from Brazil, where it was originally described, extended to the USA The fact that we detected another new species of a marsupial parasite through the use of DNA sequences clearly indicated that future studies should consider the use of molecular tools that greatly enhance our ability to delimit species, and this will increase our understanding of the species diversity of marsupial parasites.

Finally, we consider that the use of SEM is fundamental in determining reliable characters that distinguish among echinostomid species because the presence of a wide array of spines along the body and around the oral sucker. In this case, SEM was very important in showing that the species *R.macracanthus* do possess oral spines, although they are not completely visible using light microscopy (see Fig. 5C–F). For instance, Figure 5C shows the presence of 16 tongue-shaped oral spines in *R.macracanthus*, and these spines contrast in size and shape with flanking spines which are more visible. Haverkost and Gardner (2008) taxonomic key pointed out the lack of oral spines in *R.macracanthus*. However, our study demonstrated that these spines are present. Overall, the integrative taxonomy approach, where several sources of information are used to establish more robust species delimitation criteria, is highly recommended for a complete understanding of parasite diversity.

## Supplementary Material

XML Treatment for Rhopalias oochi

## Supplementary materials

Supplementary material 1 Figure S1. Tree inferred with cox1 data set, using Maximum Likelihood and Bayesian Inference Data type: phylogenetic tree Explanation note: The numbers at the nodes represent ML bootstrap support and posterior probability values. Clade terminals represent localities and hosts from the worms were collected. This dataset is made available under the Open Database License (http://opendatacommons.org/licenses/odbl/1.0/). The Open Database License (ODbL) is a license agreement intended to allow users to freely share, modify, and use this Dataset while maintaining this same freedom for others, provided that the original source and author(s) are credited.Jorge López-Caballero, Rosario Mata-López, Gerardo Pérez-Ponce De León

Supplementary material 2 Figure S2. Tree inferred with ITS region (ITS1 + 5.8S + ITS2) data set, using Maximum Likelihood and Bayesian Inference Data type: phylogenetic tree Explanation note: The numbers at the nodes represent ML bootstrap support and posterior probability values. Clade terminals represent localities and hosts from the worms were collected. This dataset is made available under the Open Database License (http://opendatacommons.org/licenses/odbl/1.0/). The Open Database License (ODbL) is a license agreement intended to allow users to freely share, modify, and use this Dataset while maintaining this same freedom for others, provided that the original source and author(s) are credited.Jorge López-Caballero, Rosario Mata-López, Gerardo Pérez-Ponce De León

Supplementary material 3 Figure S3 Data type: species images Explanation note: *Rhopaliascoronatus* ex *Didelphisvirginiana* from Los Tuxtlas, Veracruz. (**A**) Line drawing, ventral view (**B**) Microphotograph, ventral view of entire body. *Rhopaliasmacracanthus* ex *Philanderopossum* from Tlacotalpan, Veracruz (**C**) Line drawing, ventral view. (**D**) Microphotograph, ventral view of entire body. Scale bars 1 mm (**A, C**); 20.2 mm (**B, D**). This dataset is made available under the Open Database License (http://opendatacommons.org/licenses/odbl/1.0/). The Open Database License (ODbL) is a license agreement intended to allow users to freely share, modify, and use this Dataset while maintaining this same freedom for others, provided that the original source and author(s) are credited.Jorge López-Caballero, Rosario Mata-López, Gerardo Pérez-Ponce De León

## References

[B1] Acosta-Virgen K López-Caballero J García-Prieto L Mata-López R (2015) Helminths of three species of opossums (Mammalia, Didelphidae) from Mexico. ZooKeys 511: 131–152. 10.3897/zookeys.511.9571 PMC4523750 26257556

[B2] Adams BJ (2002) The species delimitation uncertainty principle. Journal of Nematology 33: 153–160. PMC2620516 19265874

[B3] Blouin MS (2002) Molecular prospecting for cryptic species of nematodes: mitochondrial DNA versus internal transcribed spacer. International Journal for Parasitology 32: 527–531. 10.1016/S0020-7519(01)00357-5 11943225

[B4] Bowles J McManus DP (1993) Rapid discrimination of *Echinococcus* species and trains using a PCR-based method. Molecular and Biochemical Parasitology 57: 231–239. 10.1016/0166-6851(93)90199-8 8094539

[B5] Bowles J Blair D McManus DP (1995) A molecular phylogeny of the human schistosomes. Molecular Phylogenetics and Evolution 4: 103–109. 10.1006/mpev.1995.1011 7663756

[B6] Braun M (1901) Zur Kenntnis der Säugetiere. Zoologische Jahrbücher Systematik 14: 311–348.

[B7] Cervantes FA Arcangeli-Álvarez J Hortelano-Moncada Y Borisenko AV (2010) DNA barcodes effectively identify the morphologically similar Common Opossum (*Didelphismarsupialis*) and Virginia Opossum (*Didelphisvirginiana*) from areas of sympatry in Mexico. Mitochondrial DNA 21: 44–50. 10.3109/19401736.2010.538051 21271858

[B8] Chandler AC (1932) Notes on helminth parasites of the opossum (*Didelphisvirginiana*) in Southeast Texas with description of four new species. Proceedings of the United States National Museum 81: 1–15. 10.5479/si.00963801.81-2939.1

[B9] Criscione CD Poulin R Blouin MS (2005) Molecular ecology of parasites: elucidating ecological and microevolutionary processes. Molecular Ecology 14: 2247–2257. 10.1111/j.1365-294X.2005.02587.x 15969711

[B10] Diesing CM (1850) Systema helminthum. Vindobonae, Vienna, 588 pp.

[B11] Drummond AJ Ashton B Buxton S Cheung M Cooper A Duran C Field M Heled J Kearse M Markowitz S Moir R Stones-Havas S Sturrock S Thierer T Wilson A (2010) Geneious v5.0.4. http://www.geneious.com/

[B12] García-Prieto L Falcón-Ordaz J Guzmán-Cornejo C (2012) Helminth parasites of wild Mexican mammals: list of species, hosts and geographical distribution. Zootaxa 3290: 1–92. 10.11646/zootaxa.3290.1.1

[B13] Haverkost TR Gardner SL (2008) A review of species in the genus *Rhopalias* (Rudolphi, 1819). Journal of Parasitology 94: 716–726. 10.1645/GE-1423.1 18605801

[B14] Hernández-Mena DI García-Prieto L García-Varela M (2014) Morphological and molecular differentiation of Parastrigea (Trematoda: Strigeidae) from Mexico, with the description of a new species. Parasitology International 63: 315–323. 10.1016/j.parint.2013.11.012 24309555

[B15] Kifune T Uyema N (1982) Report of the Fukuoka University Scientific Expedition to Peru, 1976. Part 3. Taxonomical studies on trematodes from marsupials and rodents with records of two crabs. Medical Bulletin of Fukuoka University 9: 241–256.

[B16] Kostadinova A (2005) Family Echinostomatidae Looss, 1899. In: Jones A Bray RA Gibson DI (Eds) Keys to the Trematoda, vol.2. CABI. Publishing and the Natural History Museum, Wallingford and London, UK, 9–64. 10.1079/9780851995878.0009

[B17] Looss A (1899) Weitere Beiträge zur Kenntnis der Trematoden-Fauna Aegyptens zugleich Versuch einer natürlichen Gleiderung des Genus *Distomum* Retzius. Zoologische Jahrbücher. Abteilung für Systematik, Geographie und Biologie der Tiere 12: 521–784. 10.5962/bhl.part.2037

[B18] López-Caballero J Mata-López R García-Varela M Pérez-Ponce de León G (2015) Genetic variation of *Oligacanthorhynchusmicrocephalus* (Acanthocephala: Archiacanthocephala: Oligacanthorhynchidae), parasite of three species of opossums (Mammalia: Didelphidae) across central and southeastern Mexico. Comparative Parasitology 82: 175–186. 10.1654/4742.1

[B19] Maddison DR Maddison WP (2011) Mesquite: a modular system for evolutionary analysis v2.75 http://mesquiteproject.org

[B20] Messing J (1993) M13 cloning vehicles. Their contribution to DNA sequencing. Methods in Molecular Biology 23: 9–22. 10.1016/0076-6879(83)01005-8 8220775

[B21] Moszczynska A Locke SA McLaughlin JD Marcogliese DJ Crease TJ (2009) Development of primers for mitochondrial cytocrome *c* oxidase I gene in digenetic trematodes (Platyhelminthes) illustrates the challenge of barcoding parasitic helminths. Molecular Ecology Resources 9: 75–82. 10.1111/j.1755-0998.2009.02634.X 21564967

[B22] Nadler SA (2002) Species delimitation and nematode biodiversity: phylogenies rule. Nematology 4: 615–625. 10.1163/15685410260438908

[B23] Nadler SA Pérez-Ponce de León G (2011) Integrating molecular and morphological approaches for characterizing parasite cryptic species: implications for parasitology. Parasitology 138: 1688–1709. 10.1017/S003118201000168X 21281559

[B24] Pleijel F Jondelius U Norlinder E Nygren A Oxelman B Schander C Sundberg P Thollesson M (2008) Phylogenies without roots? A plea for the use of vouchers in molecular phylogenetic studies. Molecular Phylogenetics and Evolution 48: 369–371. 10.1016/j.ympev.2008.03.024 18424089

[B25] Posada D Crandall KA (1998) Modeltest: testing the model of DNA substitution. Bioinformatics 9: 817–818. 10.1093/bioinformatics/14.9.817 9918953

[B26] Rambaut A (2006) FigTree v1.3.1. Institute of Evolutionary Biology. University of Edinburgh, UK.

[B27] Rivillas C Caro E Carvajal H Vélez I (2004) Algunos trematodos digeneos (Rhopaliasidae, Opistorchiidae) de *Philanderopossum* (Marsupialia) de la costa pacífica colombiana, incluyendo *Rhopaliascaucensis* sp. n. Revista de la Academia Colombiana de Ciencias Exactas, Físicas y Naturales 28: 591–600.

[B28] Ronquist F Teslenko M Van Der Mark P Ayres LD Darling A Höhna S Larget B Liu L Suchard MA Huelsenbeck JP (2012) MrBayes v3.2: Efficient Bayesian Phylogenetic Inference and Model Choice Across a Large Model Space. Systematics Biology 61: 539–542. 10.1093/sysbio/sys029 PMC3329765 22357727

[B29] Rudolphi CA (1809) Entozoorum sive vermium intestinalium. Historia Naturalis 1: 1–527.

[B30] Rudolphi CA (1819) Entozoorum synopsis cui accedunt mantissa duplex et indices locupletissimi. Sumtibus Augusti Rücker, Berolini (Berlin), 811 pp 10.5962/bhl.title.9157

[B31] Saijuntha W Sithithaworn P Duenngai K Kiatsopit N Andrews RH Petney TN (2011) Genetic variation and relationships of four species of medically important echinostomes (Trematoda: Echinostomatidae) in South-East Asia. Infection, Genetics and Evolution 11: 375–381. 10.1016/j.meegid.2010.11.009 21129506

[B32] Schmidt GD (1972) Revision of the Class Archiacanthocephala Meyer, 1931 (Phylum Acanthocephala), with emphasis on Oligacanthorhynchidae Southwell et MacFie, 1925. Journal of Parasitology 58: 290–297. 10.2307/3278091 5022866

[B33] Stamatakis A (2006) RAxML-VI-HPC: maximum likelihood-based phylogenetic analyses with thousands of taxa and mixed models. Bioinformatics 22: 2688–90. 10.1093/bioinformatics/btl446 16928733

[B34] Stiles CW Hassall A (1898) Notes on parasites, an inventory of the genera and subgenera of the trematode family Fasciolidae. Archives de Parasitologie 1: 81–99.

[B35] Tamura K Stecher G Peterson D Filipski A Kumar S (2013) MEGA6: Molecular Evolutionary Genetics Analysis v6.0. Molecular Biology and Evolution 30: 2725–29. 10.1093/molbev/mst197 24132122 PMC3840312

[B36] Thompson J Higgins D Gibson T (1994) Clustal W: improving the sensitivity of progressive multiple sequence alignment through sequence weighting, position-specific gap penalties and weight matrix choice. Nucleic Acids Research 22: 4673–4680. 10.1093/nar/22.22.4673 7984417 PMC308517

[B37] Tkach VV Kudlai O Kostadinova A (2016) Molecular phylogeny and systematics of the Echinostomatoidea Looss, 1899 (Platyhelminthes: Digenea). International Journal for Parasitology 46: 171–185. 10.1016/j.ijpara.2015.11.001 26699402

[B38] Vilas R Criscione CD Blouin MS (2005) A comparison between mitochondrial DNA and the ribosomal internal transcribed regions in prospecting for cryptic species of platyhelminth parasites. Parasitology 131: 1–8. 10.1017/S0031182005008437 16336737

