# Supplementary figures and images for: Molecular data reveal a new species of Rhopalias Stiles & Hassall, 1898 (Digenea, Echinostomatidae) in the Common opossum, Didelphismarsupialis L. (Mammalia, Didelphidae) in the Yucatán Peninsula, Mexico
Source: Zookeys. 2019 Jun 10;854:145–63. doi: 10.3897/zookeys.854.34549 (PMC6580842; doi:10.3897/zookeys.854.34549)

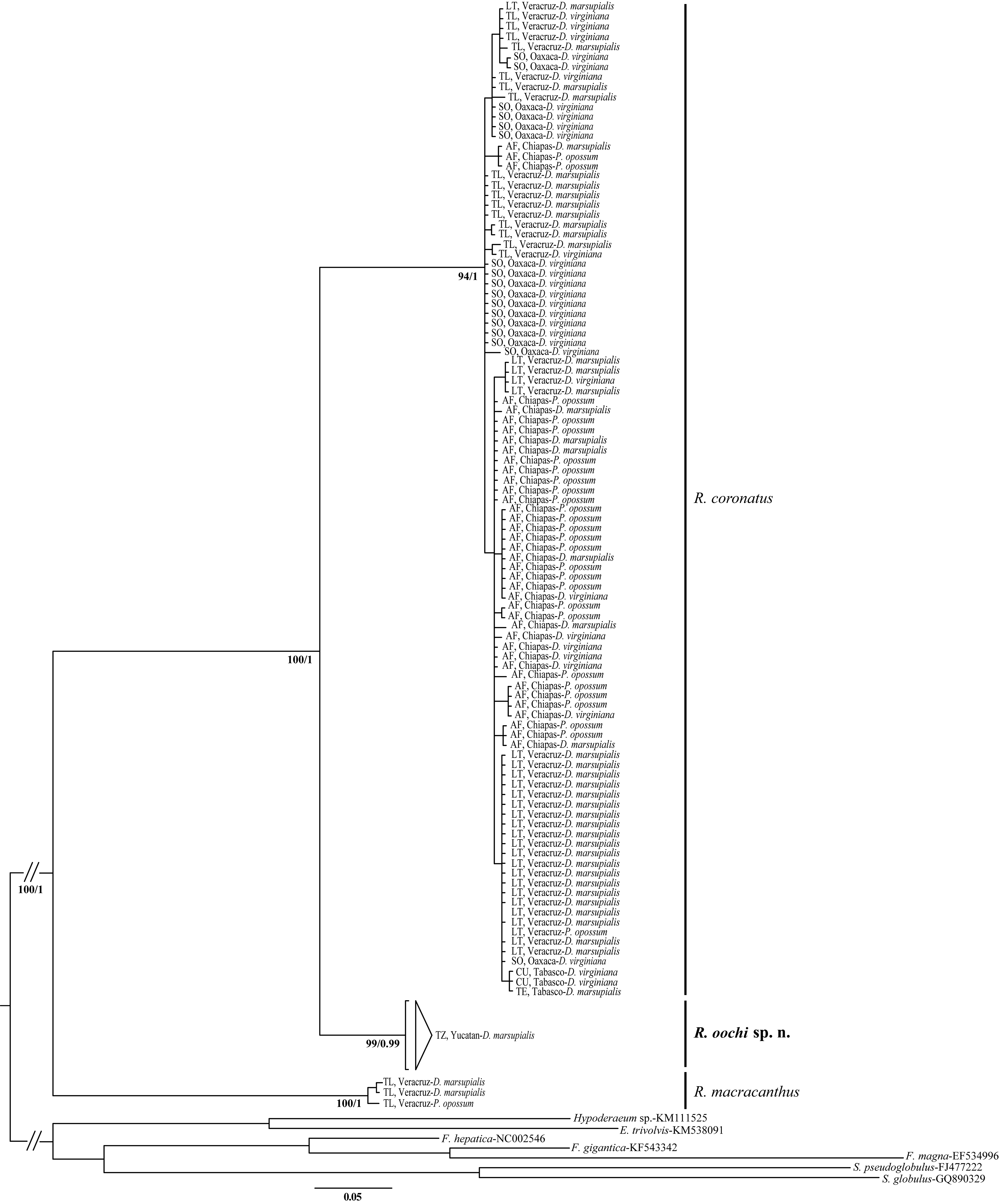

Supplement: Supplementary material 1 [file zookeys-854-145-s001.tif]

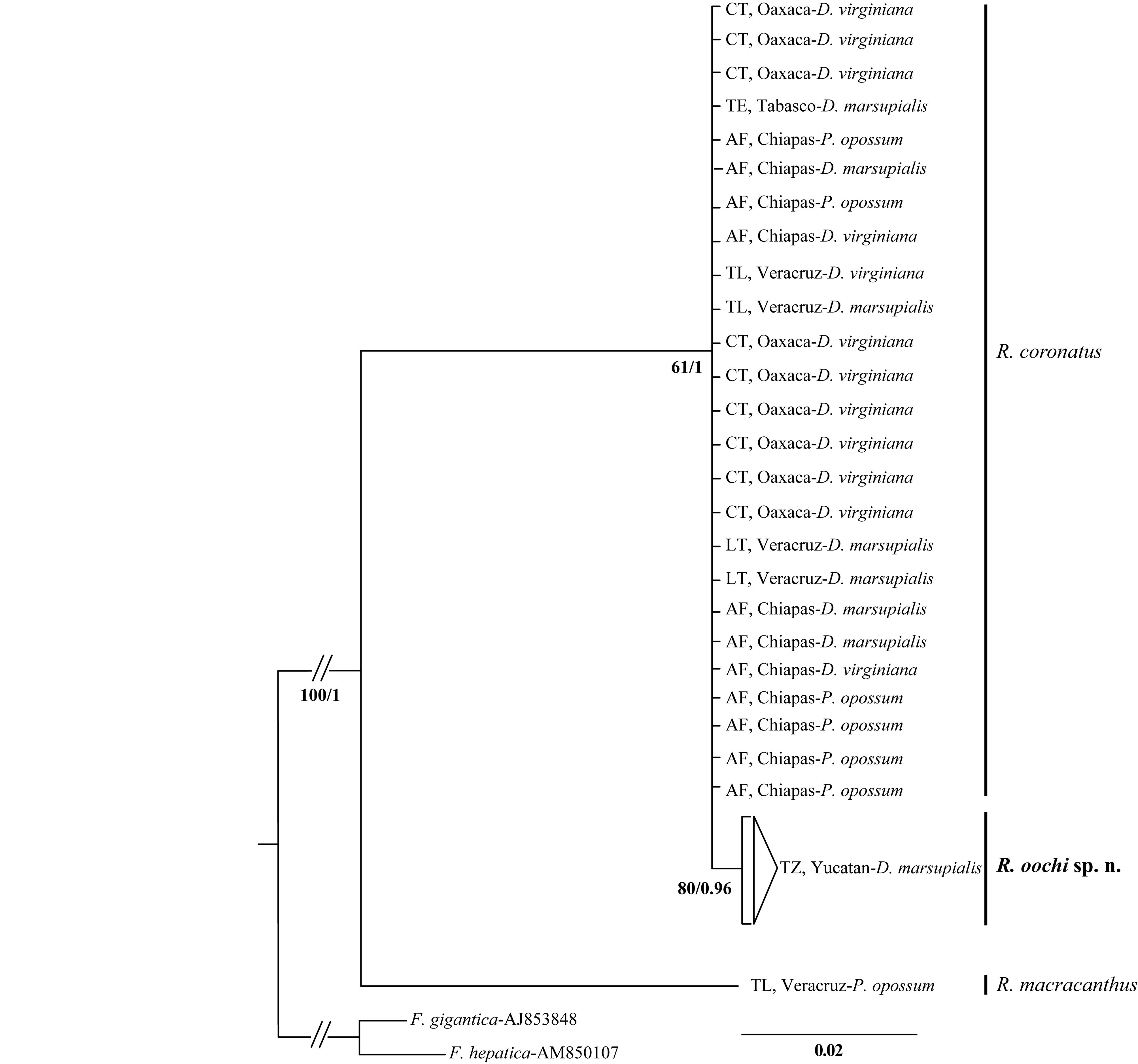

Supplement: Supplementary material 2 [file zookeys-854-145-s002.tif]

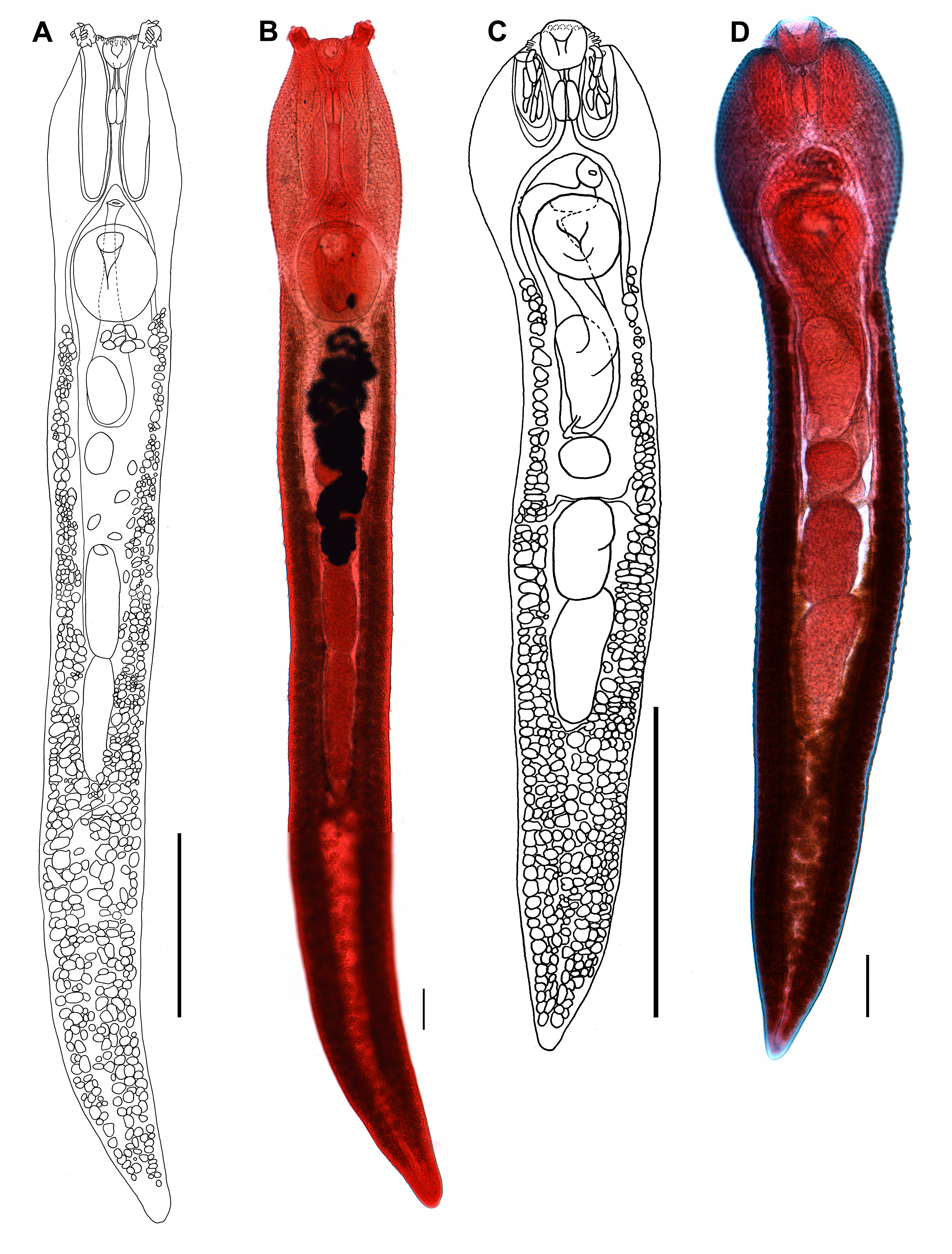

Supplement: Supplementary material 3 [file zookeys-854-145-s003.tif]
